# A retrospective study on a nomogram combining clinical and ultrasound parameters for differentiating solitary parathyroid adenoma from carcinoma or atypical tumors

**DOI:** 10.3389/fendo.2025.1538361

**Published:** 2025-04-04

**Authors:** Chunrui Liu, Mingxia Li, Wenxian Li, Haiyan Xue, Yidan Zhang, Shuping Wei, Jian He, Jing Yao, Zhengyang Zhou

**Affiliations:** ^1^ Department of Ultrasound, Nanjing Drum Tower Hospital, Affiliated Hospital of Medical School, Nanjing University, Nanjing, Jiangsu, China; ^2^ Department of Nuclear Medicine, Nanjing Drum Tower Hospital, Affiliated Hospital of Medical School, Nanjing University, Nanjing, China; ^3^ Department of Radiology, Nanjing Drum Tower Hospital, Affiliated Hospital of Medical School, Nanjing University, Nanjing, China

**Keywords:** parathyroid neoplasms, parathyroid carcinoma, ultrasonography, nomograms, primary hyperparathyroidism

## Abstract

**Objective:**

Parathyroid carcinoma (PC) and atypical parathyroid tumor (APT) are rare malignant parathyroid disorders with varying degrees of recurrence risk. The aim of this study was to determine an effective model for discriminating PC/APT among solitary parathyroid lesions.

**Methods:**

A total of 439 patients with histologically confirmed primary hyperparathyroidism were retrospectively enrolled. The training cohort comprised 207 patients, the validation cohort comprised 52 patients from Hospital I, and the external validation cohort comprised 180 patients from Hospital II. All patients were diagnosed in the parathyroid adenoma (PA) group and the APT/PC group. The clinical and ultrasonic features of the two patient groups were compared. Multivariate logistic regression analysis was conducted to identify independent risk factors for APT/PC. A nomogram was built based on multivariate logistic regression analysis. Model discrimination was assessed using receiver operating characteristic (ROC) curve analysis. The area under the curve (AUC), sensitivity, specificity, and accuracy were reported. Decision and calibration curve analyses were performed to assess the clinical value and calibration of each model, respectively.

**Results:**

In the training set, there were 181 cases of PA and 26 cases of APC/PC. Intact parathyroid hormone (iPTH) [odds ratio (OR): 1.019, 95% confidence interval (CI): 1.008–1.032], shape (OR: 16.625, 95% CI: 5.922–51.883), and relation with the thyroid capsule (OR: 3.422, 95% CI: 1.455–9.152) were independent predictive factors associated with the risk of APT/PC. The AUCs for training and internal and external validation were 0.929, 0.962, and 0.965, respectively. The accuracy, sensitivity, and specificity were 86%, 96%, and 85% in the training cohort; 92%, 100%, and 90% in the validation cohort; and 88%, 100%, and 88% in the external validation cohort, respectively. In addition, calibration plots graphically showed good agreement in the presence of the APT/PC group between risk estimation by the nomogram and histopathologic confirmation of surgical specimens. DCA in the current study showed that the nomogram was more effective than all-patient treatment or no treatment over a wide range of threshold probabilities.

**Conclusions:**

Ultrasonic features in combination with iPTH levels may be an applicable model for predicting potentially malignant parathyroid tumors and has a better potential to facilitate preoperative decision-making.

## 1 Introduction

Parathyroid neoplasms are a heterogeneous group of tumors affecting 0.1%–5.0% of the global population (1). Patients often present with hyperparathyroidism and hypercalcemia. The 2022 World Health Organization (WHO) classification classifies parathyroid neoplasms into parathyroid adenoma (PA), atypical parathyroid tumors (APTs), and parathyroid carcinoma (PC) (2). Approximately 85% of patients harbor a single PA and can be treated conservatively or with minimally invasive parathyroidectomy (MIP) (3). APT and PC are comparatively rare, comprising 0.5%–5% of patients in Western countries but up to 6%–11.5% in Asian countries (4–6). *En bloc* resection, but not local excision, is often required for APT and PC (7). APT and PC exhibit similar aggressive characteristics in routine histopathology. However, APT lacks the complete capsular or vascular invasion observed in PC (8). Clinically, both APT and PC patients require frequent follow-up to detect regional or distant metastasis (9). Approximately 60% of PC patients face repeated recurrence or metastasis, leading to fatal hypercalcemia (4, 10). Preoperative identification of such aggressive parathyroid tumors and extensive neck surgery is important for patient prognosis.

Typical PA, APT, or PC exhibits similar or overlapping clinical symptoms. These symptoms include nephrolithiasis, bone and joint pain, fatigue, weakness, anxiety, and mood disturbances. However, some individuals may be asymptomatic (5). High parathyroid hormone (PTH), alkaline phosphatase (ALP), and 24-h urinary calcium excretion levels may be predictive of carcinoma (11). Schulte et al. found that parathyroid lesions <3 cm in patients with serum calcium levels <3 mmol/L are rarely malignant if other cancer-indicative features are absent (7). However, relying solely on the preoperative biochemical or clinical manifestations makes preoperative differentiation challenging. Moreover, preoperative biopsy is not recommended for parathyroid neoplasms because of the risk of neoplastic cells and influence of surgical pathology (4, 7). Hence, there is an urgent need for precise and efficient imaging markers for the preoperative classification of malignant or potentially malignant lesions.

Cervical ultrasonography (US) and [99mTc] Tc-MIBI scintigraphy are recommended for localizing parathyroid neoplasms and determining the optimal surgical approach (12). High-resolution ultrasound imaging is highly accessible, is cost-effective, and does not involve exposure to radiation. Certain US characteristics may facilitate preoperative identification of malignant lesions, including irregular margins, heterogeneous echotexture, intranodular calcifications, indistinct capsule boundaries, and chaotic and heterogeneous vasculature (13). However, the value of ^99m^Tc sestamibi (MIBI) in distinguishing benign from malignant parathyroid lesions is limited because of its lack of specificity (14). Computed tomography (CT) scans help detect parathyroid masses, infiltration of surrounding structures, and distant metastases (15). [^18^F]-Fluorocholine PET/CT (FCH-PET) has the best advantage of localizing hyperfunctional parathyroids (16); however, the value of the differential diagnosis of parathyroid lesions is unknown. Thus, compared with other diagnostic techniques, ultrasound is a promising tool for predicting the potential malignancy of parathyroid lesions.

Several studies have developed ultrasound methods to predict the potential malignancy of parathyroid lesions (17–19). Liu et al. found (17) that DR (two diameters’ ratio of the lesion) and tumor infiltration in conjunction with intact parathyroid hormone (iPTH) level were independent predictors of PC. Liu et al. (18) also found that the “colored lesion” and “stiff rim” patterns on the elastogram are more indicated in PC and APT. Zhou et al. (19) developed an explainable machine learning model for the identification of hyper-functioning parathyroid glands. These studies are frequently constrained by their limited sample sizes and single-center data collection, as well as the utilization of sophisticated machine learning and elastography techniques, which impede the broad generalization of their findings. Herein, we conducted a retrospective study employing available clinical and ultrasonographic features and developed a predictive nomogram to differentiate PA from APT/PC in solitary parathyroid neoplasms.

## 2 Materials and methods

### 2.1 Patients

**Figure 1:**
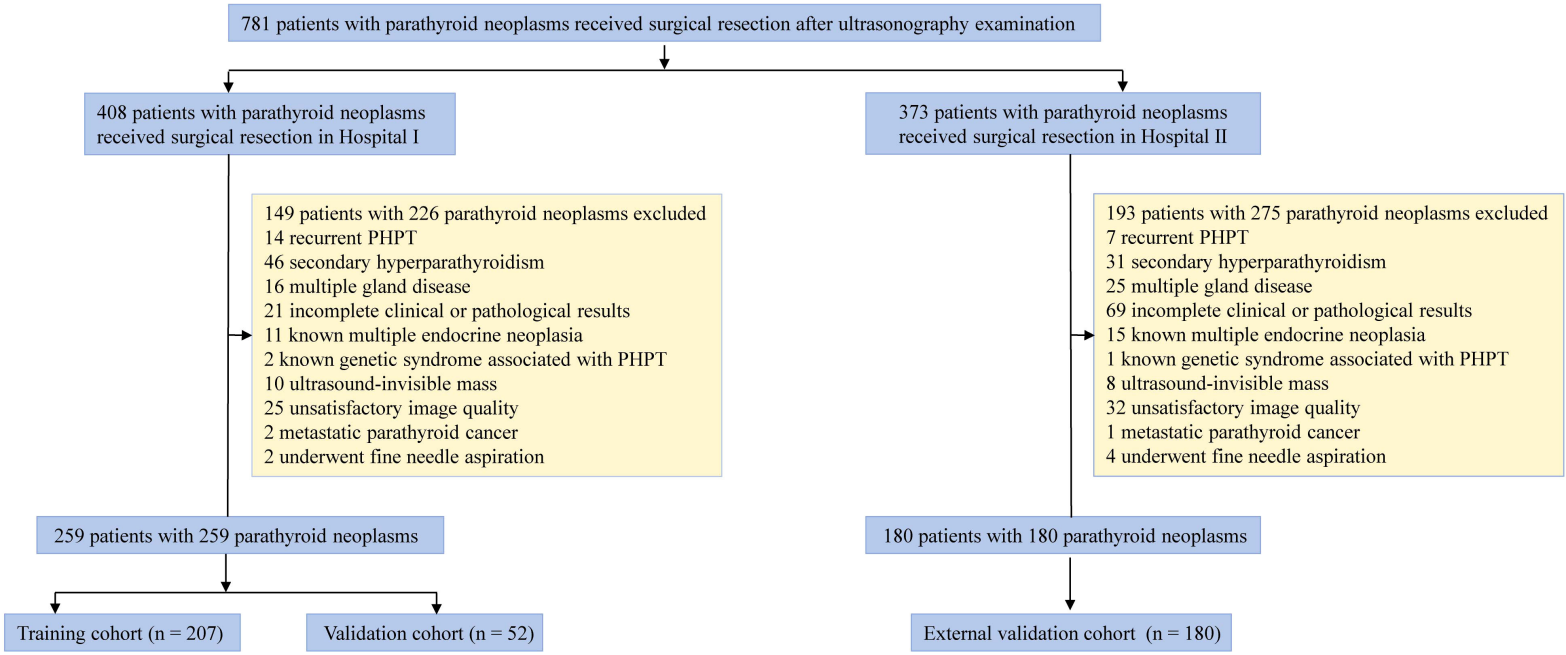
A flowchart outlining the study design. PHPT, primary hyperparathyroidism.

This retrospective study enrolled a total of 781 patients with pathologically confirmed parathyroid gland lesions, drawn from two institutions: 408 patients from institution 1 and 373 patients from institution 2. The inclusion criteria were as follows: (a) primary hyperparathyroidism (PHPT) diagnosis required persistent hypercalcemia and PTH elevation after 25OHD normalization (>30 ng/mL for ≥4 weeks); (b) US examination before surgery; (c) bilateral neck exploration (BNE) and MIP; (d) availability of complete clinical data; and (e) pathologically confirmed diagnosis of solitary PA, APT, or PC by a pathologist using the 2022 WHO criteria (2) and a follow-up period of at least 6 months. Exclusion criteria were as follows: (a) recurrent PHPT, secondary hyperparathyroidism (SHPT), multiple gland disease, and known multiple endocrine neoplasia/known genetic syndrome associated with PHPT; (b) absence of clinical and ultrasonic data; (c) an unclear pathological diagnosis; (d) patients with ultrasound-invisible masses; (e) unsatisfactory image quality for analysis, such as the presence of marks or artifacts in the US images; (f) patients with metastatic parathyroid cancer; and (g) patients who underwent fine-needle aspiration. The study protocol was approved by the ethics committee of the participating hospital (2024-611-01) and adhered to the principles outlined in the Declaration of Helsinki and the Good Clinical Practice guidelines (27). The requirement for informed consent from patients was waived. A flowchart outlining the study design is shown in **Figure 1**.

In total, 439 patients with histologically confirmed PHPT from two hospitals were included in this study between 1 January 2016 and 30 June 2024. A total of 259 patients from Hospital I (Nanjing Drum Tower Hospital) were reviewed. Patients were randomly divided into a training set and an internal validation set at a ratio of 7:3. A total of 207 and 52 patients were identified as the training and validation cohorts, respectively. A total of 180 patients from Hospital II (First Affiliated Hospital of Nanjing Medical University) were recruited as the external validation cohort.

### 2.2 Clinical parameters

The preoperative clinical and biochemical parameters collected for analysis included age, sex, preoperative serum iPTH, and serum calcium. Serum iPTH was quantified using standardized second-generation chemiluminescent immunoassays (Siemens Centaur XP or Atellica IM platforms) targeting full-length PTH (1–84) in two hospitals. We carefully searched for the maximal calcium with concomitant PTH levels at the individual level in the patients’ charts. Abnormal values were defined according to laboratory-specific reference ranges. The normal range for serum calcium at our institute is 2.25–2.75 mmol/L and that for serum iPTH is 1.31–8.14 pmol/L.

### 2.3 US examination and interpretation

All US examinations were conducted by four board-certified radiologists with more than 5 years of experience in superficial tissue ultrasound imaging. The equipment used included LOGIQ E9 (GE Healthcare, USA), EPIQ 5 (Philips, Netherlands), and Resona 7 (Mindray, China), with high-frequency probes. Image settings such as time-gain compensation, focal position, and dynamic range were optimized according to the manufacturer’s guidelines. The patients were examined in the supine position with neck hyperextension. Scanning started from the thoracic inlet and the base of the common carotid artery origins, proceeding upward to the carotid bifurcation and submental region, including the lateral cervical areas. US images were captured in transverse and longitudinal sections, with the largest horizontal and vertical cross-sections recorded for analysis. Vascular supply was assessed using color Doppler flow imaging (CDFI).

**Figure 2:**
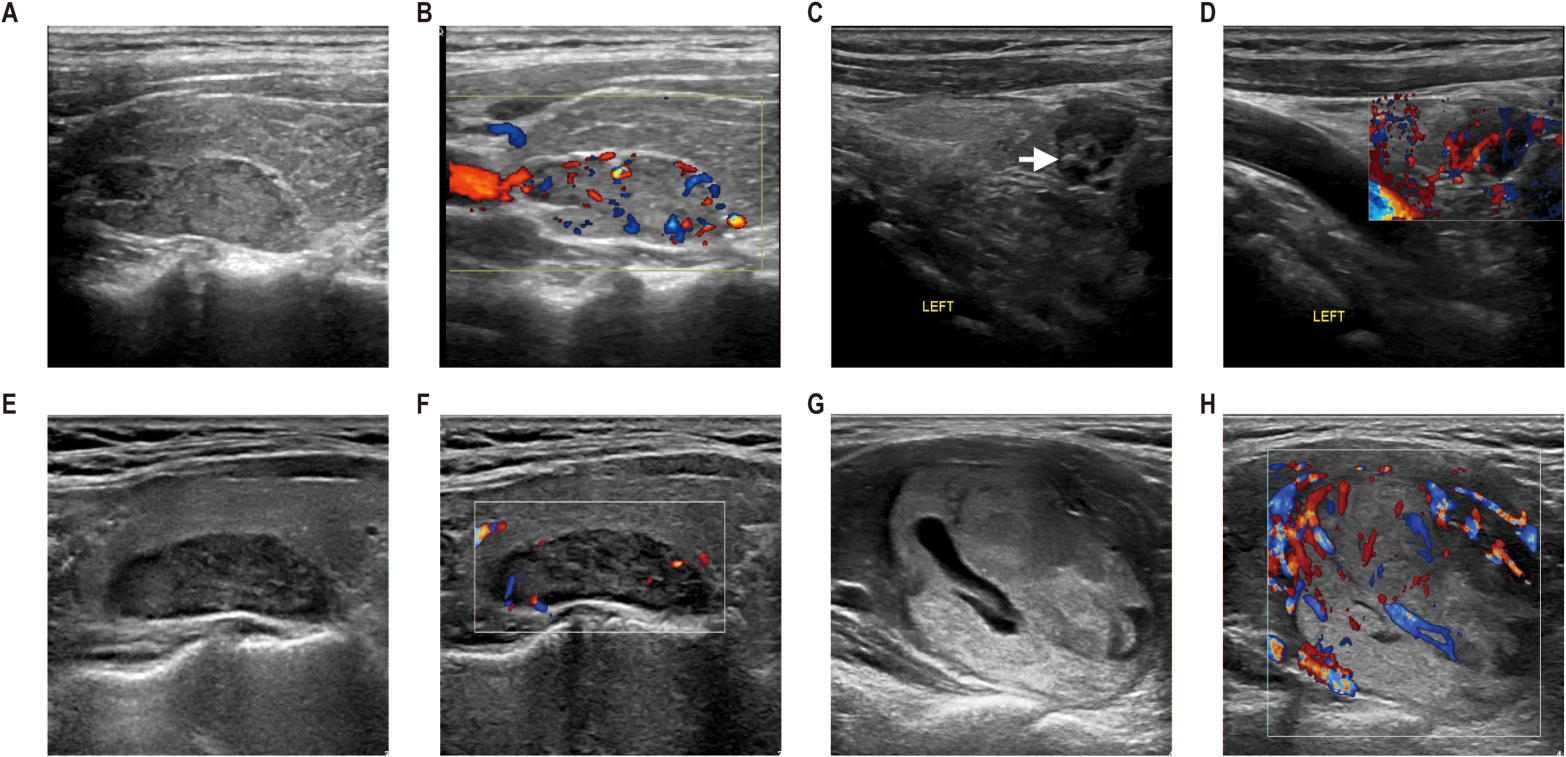
The ultrasonographic manifestations of parathyroid lesions with different pathological types. **(A, B)** Ultrasonography from a 27-year-old man with parathyroid carcinoma (PC). An isoechoic solid lesion with an incomplete capsule and irregular shape was located in the right upper parathyroid region, with the relation to the thyroid gland extending >50%. Color Doppler flow imaging (CDFI) revealed polar vessels with increased mixed vascularity. **(C, D)** Ultrasonography of a 54-year-old woman with APT. A hypoechoic solid lesion with incomplete capsule, irregular shape, and internal calcification (arrows) was observed in the lower left parathyroid region, with the relation to the thyroid gland extending ≤50%. CDFI demonstrated polar vessels with increased mixed vascularity. **(E, F)** Ultrasonography of a 51-year-old woman with parathyroid adenoma. Ultrasound examination revealed a hypoechoic lesion with complete capsule and regular shape in the upper left parathyroid region. The relation to the thyroid gland extended >50%. CDFI, avascularity. **(G, H)** Ultrasonography of a 49-year-old woman with parathyroid adenoma. A mixed echotexture lesion with cystic change <50% was identified in the lower left parathyroid region, exhibiting a complete capsule and regular shape. The relation to the thyroid gland extended >50%. Postoperative pathology confirmed parathyroid adenoma.

Two experienced radiologists (6 and 8 years, respectively) reviewed all US images; they did not participate in the image acquisition and they were blinded to clinical information and final diagnoses of each patient. Ultrasound characteristics such as size, echogenicity, shape, location, composition, vascular pattern, and visualization of the polar artery were evaluated in concordance with previous studies (20, 21). The criteria used for categorizing ultrasound features are shown in **Supplementary Table S1** and **Figure 2**. A total of 50 parathyroid ultrasound images were randomly extracted. Interobserver agreement and intraobserver agreement in the radiological features were calculated to assess feature reproducibility.

### 2.4 Statistical analysis

Statistical analysis was performed by using SPSS (version 23.0) and R software (version 3.4.4). Categorical variables were compared using the chi-square test or Fisher’s exact test, and continuous variables were compared using the Student’s *t*-test or the Mann–Whitney *U* test. Interobserver agreement in the radiological features was calculated using weighted kappa statistics for categorical variables and ICC to assess feature reproducibility. An ICC value > 0.75 was regarded as good agreement. Variables with significant differences between PA and APT/PC in the univariate analysis were subsequently included in the multivariate logistic regression analysis. Backward stepwise factor selection was performed using the Akaike information criterion. A nomogram was built based on multivariate logistic regression analysis as a graphical presentation. Model discrimination was assessed using receiver operating characteristic (ROC) curve analysis. The area under the curve (AUC), sensitivity, specificity, and accuracy were reported. Model fit was assessed using the Hosmer-Lemeshow goodness-of-fit test. Decision and calibration curve analyses were performed to assess the clinical value and calibration of each model, respectively. Two-tailed *p* < 0.05 denoted a significant difference.

## 3 Results

### 3.1 Baseline characteristics

**Table 1** summarizes the clinical parameters, ultrasound features, and pathological subtypes of 439 patients with parathyroid neoplasms from the two hospitals. The patients were classified into three cohorts, which consisted of a training cohort (*n* = 207), a validation cohort (*n* = 52), and an external validation cohort (*n* = 180). The rates of APT/PC in the training and validation cohorts [12.6% (26/207) and 21.1% (11/52), respectively] were not significantly different (*p* = 0.114). Vascular pattern and capsule showed a significant difference between the training and validation cohorts (*p* = 0.034, 0.010), and other indicators showed no difference between the two groups (*P* > 0.05). The ultrasound features showed good reproducibility and stability (ICC > 0.75), as described in **Supplementary Table S1**.

**Table 1:** Participant baseline characteristics of the three cohorts.

Characteristic	Training cohort (*n* = 207)	Validation cohort (*n* = 52)	External validation cohort (*n* = 180)
Sex, *n* (%)			
Male	60 (29.0%)	11 (21.2%)	74 (41.1%)
Female	147 (71.0%)	41 (78.8%)	106 (58.9%)
Age at diagnosis, years	57.0 (20.0, 83.0)	54.0 (25.0, 80.0)	55.0 (25.0, 80.0)
Serum iPTH, pmol/L [M (Q_1_, Q_3_)]	22.5 (14.3, 33.1)	19.0 (8.7, 140.3)	12.3 (6.5, 212.0)
Serum calcium, mmol/L [M (Q_1_, Q_3_)]	2.9 (2.7, 3.1)	2.9 (2.5, 4.6)	3.0 (2.1, 4.8)
Size, *n* (%)			
<3 cm	149 (72.0%)	38 (72.1%)	131 (67.8%)
≥3 cm	58 (28.0%)	14 (26.9%)	49 (27.2%)
Echo texture, *n* (%)			
Hypoechoic	155 (74.9%)	40 (76.9%)	151 (83.9%)
Hyperechoic	8 (3.9%)	3 (5.8%)	1 (0.6%)
Mixed	44 (21.3%)	9 (17.3%)	28 (15.6%)
Capsule, *n* (%)			
Complete	137 (66.2%)	44 (84.6%)	169 (93.9%)
Incomplete	70 (33.8%)	8 (15.4%)	11 (6.1%)
Shape, *n* (%)			
Regular	168 (81.2%)	44 (84.6%)	152 (84.4%)
Irregular	39 (18.8%)	8 (15.4%)	28 (15.6%)
Location, *n* (%)			
Typical	193 (93.2%)	50 (96.2%)	172 (95.6%)
Ectopic	14 (6.8%)	2 (3.8%)	8 (4.4%)
Composition, *n* (%)			
Solid	181 (87.4%)	46 (88.5%)	154 (85.6%)
Cystic component ≤ 50%	22 (10.6%)	6 (11.5%)	23 (12.3%)
Cystic component > 50%	4 (1.9%)	0	3 (1.7%)
Relation with thyroid capsule, *n* (%)			
No-touching	78 (37.7%)	22 (42.3%)	109 (60.6%)
Indenting ≤ 50%	98 (47.3%)	23 (44.2%)	56 (31.1%)
Indenting > 50%	31 (15.0%)	7 (13.5%)	15 (8.3%)
Visualization of polar artery, *n* (%)			
Present	149 (72.0%)	33 (63.5%)	93 (51.7%)
None	58 (28.0%)	19 (36.5%)	87 (48.3%)
Vascular pattern, *n* (%)			
Avascular	129 (62.3%)	24 (46.2%)	71 (39.4%)
Peripheral or mixed	78 (37.7%)	28 (53.8%)	109 (60.6%)
Calcification, *n* (%)			
Present	204 (98.6%)	50 (96.2%)	172 (95.6%)
None	3 (1.4%)	2 (3.8%)	8 (4.4%)
Surgery			
MIP	169 (81.6%)	42 (80.8%)	160 (88.9%)
Bilateral neck exploration (BNE) + parathyroid adenoma excision	12 (5.8%)	3 (5.8%)	4 (2.2%)
*En bloc* resection	26 (12.6%)	7 (12.4%)	16 (8.9%)
Pathological subtype, *n* (%)			
Parathyroid adenoma	181 (87.4%)	41 (78.9%)	165 (91.7%)
Atypical parathyroid tumors	24 (11.6%)	9 (17.3%)	12 (6.7%)
parathyroid carcinoma	2 (1.0%)	2 (3.8%)	3 (1.6%)

iPTH, intact parathyroid hormone; MIP, minimally invasive parathyroidectomy; BNE, bilateral neck exploration.

### 3.2 Identification of predictive factors

The results of the univariate logistic regression analysis are presented in **Table 2**. Variables with *p* < 0.2 were PTH (*p* < 0.001), serum calcium (*p* < 0.001), size (*p* < 0.001), echo texture (*p* = 0.026), shape (*p* < 0.001), composition (*p* = 0.011), relation with thyroid capsule (*p* = 0.003), and calcification (*p* = 0.002).

**Table 2:** Univariate logistic regression analysis of potentially malignant parathyroid tumors in the training cohort.

Characteristic	PA (*n* = 181)	APT/PC (*n* = 26)	*p*-value
Sex, *n* (%)			0.258
Male	50 (27.6%)	10 (38.5%)	
Female	131 (72.4%)	16 (61.5%)	0.278
Age at diagnosis, years	57.00 (47.00, 65.00)	57.50 (37.25, 62.75)	0.519
Serum iPTH, pmol/L [M (Q_1_, Q_3_)]	19.9 (13.5, 27.7)	47.7 (32.1, 87.6)	<0.001
Serum calcium, mmol/L [M (Q_1_, Q_3_)]	2.9 (2.7, 3.1)	3.3 (3.0, 3.4)	<0.001
Size, *n* (%)			
≤3 cm	139 (76.8%)	10 (38.5%)	<0.001
≥3 cm	42 (23.2%)	16 (61.5%)	
Echo texture, *n* (%)			0.026
Hypoechoic	140 (77.3%)	15 (57.7%)	
Hyperechoic	7 (3.9%)	1 (3.8%)	
Mixed	34 (18.8%)	10 (38.5%)	
Capsule, *n* (%)			0.726
Complete	119 (65.7%)	18 (69.2%)	
Incomplete	62 (34.3%)	8 (30.8%)	
Shape, *n* (%)			<0.001
Regular	160 (88.4%)	8 (30.8%)	
Irregular	21 (11.6%)	18 (69.2%)	
Location, *n* (%)			
Typical	167 (92.3%)	26 (100%)	
Ectopic	14 (7.7%)	0	
Composition, *n* (%)			0.011
Solid	163 (90.1%)	18 (69.2%)	
Cystic component ≤ 50%	15 (8.3%)	7 (26.9%)	
Cystic component > 50%	3 (1.7%)	1 (3.8%)	
Relation with thyroid capsule, *n* (%)			0.003
No-touching	60 (33.1%)	2 (7.7%)	
Indenting ≤ 50%	92 (50.8%)	6 (23.1%)	
Indenting > 50%	29 (16.0%)	18 (69.2%)	
Visualization of polar artery, *n* (%)			0.087
Present	134 (74.0%)	15 (57.7%)	
None	47 (26.0%)	11 (42.3%)	
Vascular pattern, *n* (%)			0.343
Avascular	115 (63.5%)	14 (53.8%)	
Peripheral or mixed	66 (36.5%)	12 (46.2%)	
Calcification, *n* (%)			<0.001
Present	0	23 (88.5%)	
None	181 (100%)	3 (11.5%)	

PA, parathyroid adenoma; APT, atypical parathyroid tumor; PC, parathyroid cancer; iPTH, intact parathyroid hormone.

In the multivariate analysis, with results reported as odds ratio (OR) [95% confidence interval (CI)], PTH (1.019, 95% CI: 1.008–1.032]), shape (16.625, 95% CI: 5.922–51.883), and relation with thyroid capsule (3.422, 95% CI: 1.455–9.152) were independent predictive factors associated with the risk of potentially malignant parathyroid diseases (**Table 3**).

**Table 3:** Multivariate logistic regression analysis of potentially malignant parathyroid tumors in the training cohort.

Characteristic	Estimate	Std. Error	*z*-value	OR (95% CI)	*p*-value
iPTH	0.019	0.006	3.278	1.019 (1.008, 1.032)	0.001
Shape	2.811	0.548	5.134	16.625 (5.922, 51.883)	<0.001
Relation with thyroid capsule	1.231	0.464	2.649	3.422 (1.455, 9.152)	0.008

PTH, intact parathyroid hormone; CI, confidence interval; OR, odds ratio.

### 3.3 Nomogram construction and performance evaluation

**Figure 3:**
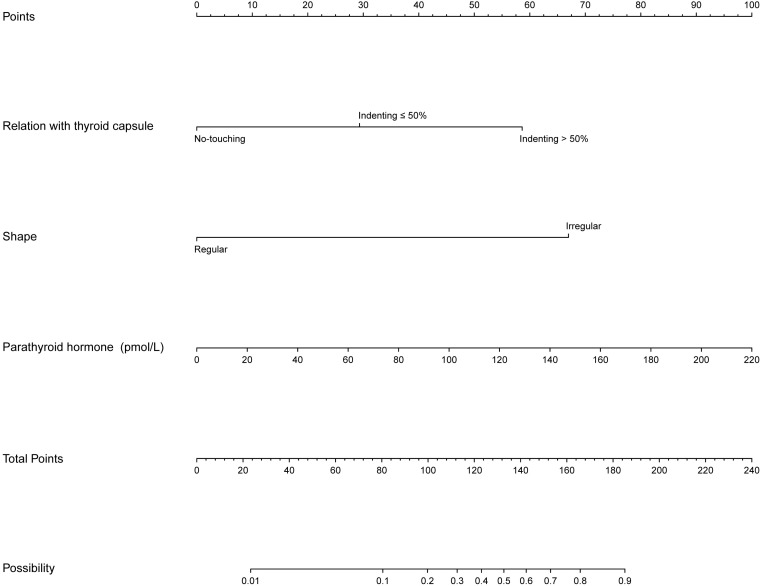
Nomogram for predicting the risk of potentially malignant parathyroid tumors.

These independent predictors were used to determine the risk of the APT/PC nomogram (**Figure 3**). To use the nomogram, the value of each patient was placed on each variable axis, and a line was drawn upward to determine the number of received points for each variable value. The sum of these numbers was located on the total point axis, and a line down the bottom axis was drawn to determine the probability of potentially malignant parathyroid tumors.

**Figure 4:**
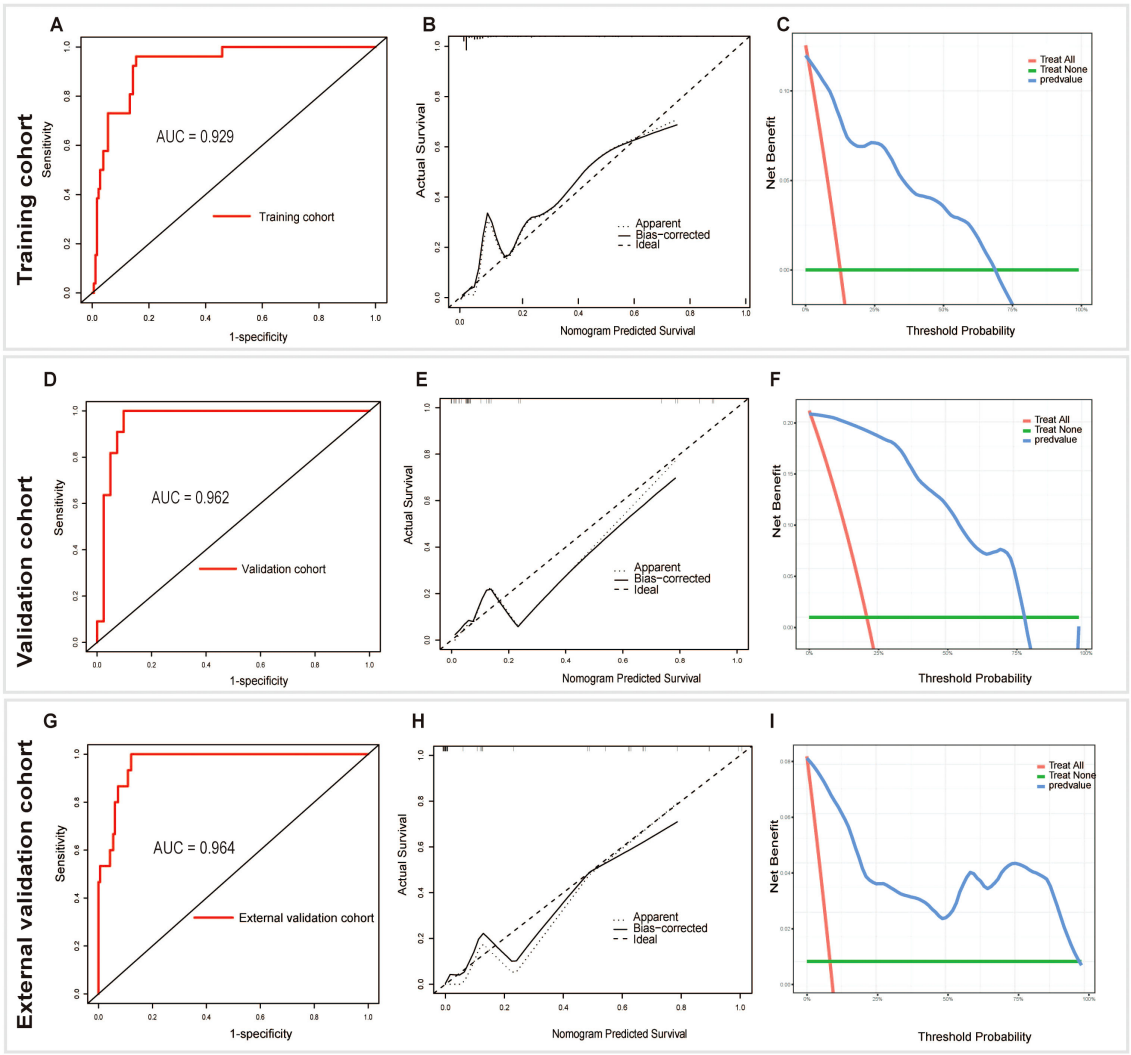
Receiver operating characteristic (AUC), calibration, and decision curve analyses (DCA) of the nomogram in the training **(A-C)**, validation **(D-F)**, and external validation **(G-I)** cohorts, respectively.

The model was validated both internally and externally by bootstrap validation. The nomogram demonstrated good accuracy for estimating the risk of aggressive parathyroid disease. The AUC for training, internal, and external validation was 0.929, 0.962, and 0.965, respectively. The accuracy, sensitivity, and specificity were 86%, 96%, and 85% in the training cohort; 92%, 100%, and 90% in the validation cohort; and 88%, 100%, and 88% in the external validation cohort, respectively (**Table 4**). In addition, calibration plots graphically showed good agreement on the presence of aggressive parathyroid diseases between risk estimation by the nomogram and histopathologic confirmation of surgical specimens. DCA in the current study showed that the nomogram of the aggressive parathyroid model used in our study was more effective than all-patient treatment or no treatment over a wide range of threshold probabilities (**Figure 4**).

**Table 4:** Accuracy of the prediction score of the nomogram for estimating the risk of aggressive parathyroid diseases.

	AUC (95% CI)	ACC (%)	SENS (%)	SPE (%)
Training cohort	0.929 (0.887, 0.973)	86 (85, 87)	96 (89, 100)	85 (79, 90)
Validation cohort	0.962 (0.913, 1.000)	92 (91, 97)	100 (96, 100)	90 (81, 99)
External validation cohort	0.965 (0.937, 0.992)	88 (82, 89)	100 (95, 100)	88 (83, 93)

AUC, area under the receiver operating characteristic curve; CI, confidence interval; ACC, accuracy; SEN, sensitivity; SPE, specificity.

## 4 Discussion

Our findings revealed three significant predictive factors: iPHT, shape, and relationship with thyroid capsule. We integrated three cohorts of patients with PHPT from two centers to develop and validate a nomogram based on the three predictive factors for parathyroid tumors. This nomogram enabled us to easily calculate the probability of APT/PC diagnosis, thereby facilitating early detection and appropriate treatment selection. This method prevents insufficient treatment, enhances patient outcomes, and minimizes the risk of complications from untreated or advanced tumors.

In our study, we identified statistically significant differences in size, echo texture, shape, composition, relation with the thyroid capsule, calcification, and iPTH and serum calcium levels between the PA and APT/PC groups (*p* < 0.05). In the APT/PC group, 61.5% of the lesions exceeded 3 cm compared to 23.2% in the PA group, which is consistent with prior research (11). Malignancies often correspond to elevated serum iPTH and calcium levels and greater tumor weight than benign conditions (7, 22, 23). In our study, the iPTH level (OR: 1.019, 95% CI: 1.008–1.032) was an independent predictive factor associated with the risk of APT/PC. However, isolated iPTH levels are not reliable predictors of malignancy, as most lesions with excessive iPTH secretion are benign. PC rarely presents as normocalcemic hyperparathyroidism (24). Borderline iPTH excess might indicate compensation for vitamin D deficiency or low calcium intake, rather than PHPT. Therefore, malignancy prediction should consider this biochemical index, along with other sonographic risk factors.

On gross examination, PA usually presents as a well-defined, smooth, red-brown nodule with a thin capsule. Ultrasound imaging revealed well-defined oval to rounded or elongated homogeneously hypoechoic nodules. However, as parathyroid lesions increase in size, echoic features can also vary. In a cohort of 907 patients with benign and atypical parathyroid adenomas, Hu et al. found an incidence of 4% for cystic parathyroid adenomas based on ultrasound or pathology. Interestingly, atypical adenomas are more common among cystic rather than solid lesions (25). The diagnosis of extensive cystic parathyroid neoplasms can be challenging because of the lack of hypersecretory behavior. These tumors may be incorrectly identified as thyroid cysts on scintigraphy, because they do not accumulate MIBI. Ultrasound is a critical diagnostic tool for differentiating between cystic and solid masses and for evaluating the risk of malignancy.

In our study, an irregular shape was observed in 69.2% of the patients in the PC/APT group and in 11.6% of the patients in the PA group. The irregular shapes include major lobulations, margin irregularities, and triangles. Shape (OR: 16.625, 95% CI: 5.922–51.883) was identified as an independent predictive factor of APT/PC risk. Additionally, Liu et al. (17) suggested that the “diameter ratio” can diagnose PC with 70.0% sensitivity, 91.7% specificity, 80.8% PPV, and 85.9% NPV. However, long-term secondary or tertiary hyperparathyroidism may result in enlarged parathyroid glands with irregular contours, which could mimic invasive growth. Therefore, caution should be exercised when diagnosing PC in patients with advanced chronic renal failure.

We also found that the relation with the thyroid capsule (OR: 3.422, 95% CI: 1.455–9.152) was an independent predictive factor associated with the risk of APT/PC. The relation between the parathyroid mass and thyroid gland is close, which indirectly reflects the aggressiveness of parathyroid tumors. Malignant tumors often exhibit infiltrative growth and invade surrounding tissues. Under these circumstances, the capsular margins of both the parathyroid nodule and the adjacent thyroid are not clearly identified, often rendering a demarcation of the cleavage plane between the thyroid and parathyroid gland.

Vascular information is crucial for diagnosing parathyroid nodules, with enlarged parathyroid glands exhibiting more hypervascularity than thyroid nodules. Hunter et al. highlighted that variations in tissue vascular pathophysiological parameters are essential for accurate localization of PAs using 4D-CT imaging (26). Liu et al. enhanced polar vessel detection and blood flow signal quantification using Angio Plus ultrasound imaging (27). Their study indicated that a visible polar artery strongly suggests a parathyroid lesion characterized by peripheral and central blood flow patterns. In line with our findings, 74.0% of patients (134 of 181) in the PA group showed polar artery visualization. However, the presence of the polar artery was not unique to PA, as demonstrated by the 57.7% visualization rate (15 of 26) in the APT/PC group. Some parathyroid neoplasms may be associated with complete or incomplete encapsulation, while there were no significant differences between the two groups in terms of the capsule in our study. This may be due to the small sample size in the APT/PC group. Incomplete encapsulation may be relevant to intraoperative adhesion and invasion of the adjacent capsule. Speaking of calcification, Shah et al. found that intratumoral calcification had 100% positive predictive value to diagnose PC on PHPT (28).

In the absence of standardized clinical guidelines for preoperative risk stratification of PC in patients with PHPT, individuals undergoing localized parathyroidectomy frequently lack systematic oncological risk evaluations. This oversight may result in suboptimal surgical interventions that inadequately address malignant potential (7). Our retrospective cohort analysis of 439 patients with parathyroid pathologies identified 7 confirmed PC cases and 45 APT cases. Among these, 49 patients (11.2%) underwent *en bloc* resection due to intraoperative findings of extensive adhesions or concurrent suspicion of malignant thyroid nodules. Historically, intraoperative decision-making at our institution relied predominantly on macroscopic adhesion severity or intraoperative frozen section results suggestive of malignancy. To address this limitation, we proposed a novel risk stratification framework for parathyroid lesions, integrating clinical and ultrasonic predictors to categorize patients into distinct therapeutic cohorts. Predictive modeling via nomogram analysis demonstrated that preoperative *en bloc* resection would have been appropriately indicated in a minimum of 56/439 patients (12.8%) with suspected APT/PC, potentially reducing recurrence risk through optimized radical resection margins. However, the diagnostic performance evaluation revealed a consistent trend of modestly reduced specificity (range: 85%–90%) relative to sensitivity (96%–100%) across the three datasets, indicating a nonnegligible false-positive rate. This diagnostic asymmetry raises concerns regarding overtreatment, which may impose unnecessary morbidity and healthcare burdens on patients with indolent pathologies.

This study had several limitations. First, this retrospective study excluded recurrent PHPT and SHPT; hence, the model had potential selection bias. Second, ultrasound findings, including tumor size and margins, were subjectively assessed by a sonographer. These subjective assessments, unlike objective factors such as age and iPTH level, could significantly influence the final nomogram results, posing a critical issue for clinical application in evaluating potential tumors. To mitigate this limitation, standardized ultrasound assessment protocols were established, and interobserver agreement and intraobserver agreement in the evaluation of radiological features were calculated. Future studies could incorporate artificial intelligence-based image analysis techniques, which could potentially provide more objective and consistent evaluations of tumor characteristics. Third, the sample sizes were small across various cohorts owing to the rarity of PC or APT cases. Fourth, this study only considered the ultrasound manifestations. Prospective cohort studies with more imaging modalities (e.g., sestamibi scans, CT, or MRI), larger sample sizes, and multiple centers are required to improve diagnostic accuracy for parathyroid lesions. Fifth, serum PTH testing may have an impact on the accuracy of our PTH measurements, which could significantly influence the nomogram validity. This study spans an era of evolving PTH assay technologies. Although all measurements utilized second-generation assays targeting intact PTH (1–84), platform transitions (e.g., from Centaur XP to Atellica IM) and reagent lot variations may introduce non-quantified inter-assay variability. In fact, we are in progress to establish a prospective study of APC/PC, and the new data may shed light on the problems mentioned above.

In summary, we developed and validated a nomogram to improve preoperative diagnosis of parathyroid lesions. This diagnostic tool can aid clinicians in identifying patients at a higher risk of developing APT/PC, potentially leading to more appropriate management and follow-up strategies.

## Data Availability

The raw data supporting the conclusions of this article will be made available by the authors, without undue reservation.
