# Supplementary material for: A retrospective study on a nomogram combining clinical and ultrasound parameters for differentiating solitary parathyroid adenoma from carcinoma or atypical tumors
Source: Front Endocrinol (Lausanne). 2025 Apr 4;16:1538361. doi: 10.3389/fendo.2025.1538361 (PMC12005988; doi:10.3389/fendo.2025.1538361)
Supplement: Supplementary file 1 [file Table1.docx]

Supplemental Table 1. Diagnostic criteria of ultrasound features

| Feature name | Diagnostic criteria |
| --- | --- |
| Size | < 3 cm or ≥3 cm according to the maximum diameter(mm) on transverse images |
| Echo texture | hypoechoic, isoechoic, hyperechoic, or mixed when compared with that of the surrounding parenchyma |
| Capsule | complete or incomplete |
| Shape | regular or irregular, Oblong or oval was defined as regular；  major lobulations, margin irregularities, and triangles were recorded as irregular. |
| Location | typical location included the upper right, lower right, upper left, lower left；  ectopic regions included the intrathyroid or extrathyroid, such as those located at the mediastinum and carotid bifurcation |
| Composition | solid, visually less than 50% cystic component, and cystic component > 50%. |
| Relation with thyroid capsule | non-touching, indenting < 50%, or indenting > 50% |
| Visualization of polar artery | polar artery demonstrates vessel reaches the pole of the parathyroid gland and then divides it either in a fork pattern with sub-branches or as an arc, and then the branches supply the entire gland |
| Vascular pattern | avascularity or increased peripheral or mixed vascularity |
| Calcification | punctate echogenic foci with or without posterior shadowing |

Supplementary Table 2 Interobserver agreement and intra-observer agreement in the radiological features

| Features | Intraobserver agreement ICC value | Interobserver agreement ICC value |
| --- | --- | --- |
| Size | 0.954(0.921, 0.974) | 0.908(0.844, 0.947) |
| Echo texture | 0.846(0.744, 0.910) | 0.833(0.724, 0.902) |
| Capsule | 0.900(0.830, 0.942) | 0.856(0.760, 0.916) |
| Shape | 0.905(0.838, 0.945) | 0.861(0.767, 0.918) |
| Location | 0.802(0.673, 0.887) | 0.793(0.662, 0.877) |
| Composition | 0.941(0.898, 0.966) | 0.888(0.810, 0.935) |
| Relation with thyroid capsule | 0.869(0.780, 0.923) | 0.826(0.713, 0.898) |
| Visualization of polar artery | 0.800(0.673, 0.881) | 0.846(0.743, 0.909) |
| Vascular pattern | 0.803(0.677, 0.883) | 0.880(0.797, 0.930) |
| Calcification | 1.000 | 0.847(0.749,0.912) |
